# Deficiencies of the Lipid-Signaling Enzymes Phospholipase D1 and D2 Alter Cytoskeletal Organization, Macrophage Phagocytosis, and Cytokine-Stimulated Neutrophil Recruitment

**DOI:** 10.1371/journal.pone.0055325

**Published:** 2013-01-28

**Authors:** Wahida H. Ali, Qin Chen, Kathleen E. Delgiorno, Wenjuan Su, Jason C. Hall, Tsunaki Hongu, Huasong Tian, Yasunori Kanaho, Gilbert Di Paolo, Howard C. Crawford, Michael A. Frohman

**Affiliations:** 1 Department of Pharmacology, Stony Brook University, Stony Brook, New York, United States of America; 2 Center for Developmental Genetics, Stony Brook University, Stony Brook, New York, United States of America; 3 Graduate Program in Molecular and Cellular Pharmacology, Stony Brook University, Stony Brook, New York, United States of America; 4 Department of Physiological Chemistry, Graduate School of Comprehensive Human Sciences and Institute of Basic Medical Sciences, University of Tsukuba, Tsukuba, Japan; 5 Department of Pathology and Cell Biology, Columbia University Medical Center, New York, New York, United States of America; J. Heyrovsky Institute of Physical Chemistry, Czech Republic

## Abstract

Cell migration and phagocytosis ensue from extracellular-initiated signaling cascades that orchestrate dynamic reorganization of the actin cytoskeleton. The reorganization is mediated by effector proteins recruited to the site of activity by locally-generated lipid second messengers. Phosphatidic acid (PA), a membrane phospholipid generated by multiple enzyme families including Phospholipase D (PLD), has been proposed to function in this role. Here, we show that macrophages prepared from mice lacking either of the classical PLD isoforms PLD1 or PLD2, or wild-type macrophages whose PLD activity has been pharmacologically inhibited, display isoform-specific actin cytoskeleton abnormalities that likely underlie decreases observed in phagocytic capacity. Unexpectedly, PA continued to be detected on the phagosome in the absence of either isoform and even when all PLD activity was eliminated. However, a disorganized phagocytic cup was observed as visualized by imaging PA, F-actin, Rac1, an organizer of the F-actin network, and DOCK2, a Rac1 activator, suggesting that PLD-mediated PA production during phagocytosis is specifically critical for the integrity of the process. The abnormal F-actin reorganization additionally impacted neutrophil migration and extravasation from the vasculature into interstitial tissues. Although both PLD1 and PLD2 were important in these processes, we also observed isoform-specific functions. PLD1-driven processes in particular were observed to be critical in transmigration of macrophages exiting the vasculature during immune responses such as those seen in acute pancreatitis or irritant-induced skin vascularization.

## Introduction

Actin remodeling and reorganization of the cytoskeleton in response to signaling events initiated from the extracellular environment are necessary for morphological changes involving the plasma membrane. Actin remodeling is the end result of a tightly controlled cascade involving effectors, a variety of GTPases, and their corresponding guanine exchange factors (GEFs) [1]. The effectors, such as Arp 2/3, WAVE, and WASP, polymerize actin. Localization and regulation of the effectors is controlled by small GTPases such as Rho, Rac, and ARF. GEFs, such as DOCK2 and Vav1, exchange GDP for GTP on the GTPases, allowing them to interact with the effectors at sites of actin remodeling [2]. Coordinated recruitment and localization of effectors, GTPases, and GEFs is crucial for organized cytoskeletal movements.

Chemokines and bacteria contacting the cell surface trigger intracellular pathways through various receptors, all of which activate lipid signaling cascades [3]. Lipid signals take many forms, including phosphoinositides, phosphatidic acid (PA), and diacylglycerol (DAG), all of which can function to recruit proteins involved in cytoskeletal reorganization [4]. GEFs are well known for binding to these lipids via specific protein domains [5]. In many cases, GEFs, such as SOS and DOCK2, are recruited through combined or sequential interaction with multiple lipids. SOS, which activates Ras, is recruited to the plasma membrane via PI(4,5)P_2_ and PA [6], and DOCK2, a GEF for Rac1, is recruited by PI(3,4,5)P_3_ and PA [7]. GTPases have similarly been reported to be recruited to sites of stimulation via protein domains that bind to PA [5], [8].

PA is a pleiotropic lipid second messenger that can be produced by diacylglycerol kinases (DAGK), lysoPA acetyltransferases, and phospholipase D (PLD) family members during signaling events [9]. In addition to functioning as a lipid anchor for protein recruitment, PA can also promote negative membrane curvature, act as an activator of other signaling enzymes, and propagate signal transduction events via conversion of the PA to other signaling lipids [9]. PA production at sites of actin rearrangement may aid in the recruitment and orientation of GTPases [10], [11]; both PLD [11], [12], [13], [14], [15], [16] and DAGK [17], [18] activation during actin rearrangement have been reported. PA is thus important for actin reorganization; however the pathway(s) through which PA is generated might vary depending on the setting.

PLD has been proposed to play roles in both cell migration [19] and phagocytosis [20], [21]. During inflammation, damaged tissues release chemokines to attract white blood cells to the sites of injury [22], [23]. In response to the chemokines, macrophages and neutrophils form a leading edge that is orchestrated by lipid signals generated at the plasma membrane. Chemokine-stimulated neutrophil migration modeled *in vitro* has been shown to require PLD activity and PA production to generate a leading edge by directing DOCK2 localization and hence Rac1 activation [7]. PA may also function directly to recruit Rac1 to peripheral sites of actin remodeling in cell spreading [8], an important prerequisite to migration. Phagocytic ingestion by macrophage-like cell lines has been reported to decrease when PLD function is diminished via exposure to relatively non-specific inhibitors such as 1-butanol or is partially knocked down using RNAi [20], [21]. Phagocytosis uses actin rearrangement to engulf particles after surface receptors recognize specific motifs and trigger receptor clustering and signaling cascades [24], [25], [26]. Binding of IgG-coated particles to Fcγ receptors (FcγR) recruits the small GTPases Rac1, ARF6, and Cdc42 and their GEFs DOCK2 and Vav-1 to the plasma membrane [27], [28], all of which except Vav1 have been shown to have PLD-mediated interactions in other contexts. Locally-focused actin polymerization then re-shapes the plasma membrane to surround the beads in a zipper-like fashion to form a cup [28], [29].

Previous studies on immune cell migration and phagocytosis have been limited to immortalized cell lines, RNAi knock-down approaches, and nonspecific inhibitors. Thus, definitive roles for PLD1 and/or PLD2 in primary macrophages and neutrophils both *in culture* and *in vivo* have not been determined. In this study, we employ primary cells from PLD1 and PLD2 knockout mice [30], [31], [32] along with a selective inhibitor [33] to more thoroughly parse the function of PLDs in these processes. We show here that both isoforms do have roles in these processes; in macrophages lacking either isoform, we observed abnormalities in F-actin organization and in localization of actin reorganization proteins, as well as decreases in both phagocytosis and cell migration. Moreover, PA was still detected at the phagocytic cup even in the total absence of PLD activity, indicating that other pathways also generate PA in this setting, but nonetheless that PLD-generated PA production is critical for the integrity of the phagocytic process. Finally, we also show that PLD1 is selectively required during extravasation of macrophages from the bloodstream to sites of damage in interstitial tissues.

## Materials and Methods

### Animals (and Ethics Statement)

Gender- and age-matched *Pld1* ^−/−^ [34] and *Pld2* ^−/−^ [32] mice backcrossed more than 10 generations into C57BL/6 were used at 3–6 months of age. All mouse experiments were performed in compliance with the Stony Brook University Institutional Animal Care and Use Committee (IACUC) guidelines. The SBU IACUC committee specifically approved this study.

### Reagents

Mouse macrophage nucleofector kit (Lonza) was used to transfect EGFP-Spo-20 construct according to manufacturer's instructions. Mouse monoclonal anti-Rac1 (BD Transduction Laboratories), mouse monoclonal anti-activated Rac1 (NewEast Biosciences), and goat polyclonal anti-DOCK2 (Santa Cruz) were used with Alexa 488- and Alexa 680-conjugated secondary antibodies (BD transduction laboratories). The GST-PAK-PBD plasmid construct was provided as a kind gift from Dr. Richard Z. Lin, Stony Brook University.

### Isolation of primary macrophages

Bone marrow was extracted from femurs of 3-month old mice and macrophages derived as described previously [35], [36]. Twenty-four hours before use, the bone marrow-derived macrophages (BMDM) were seeded at 1.5×10^5^ cells/well in 24-well plates for confocal imaging or 2.5×10^6^ cells/well in 6-well plates for Western blot analysis.

### Stimulation of BMDM with latex beads opsonized with human IgG

3 µm latex beads (Sigma) opsonized with human IgG (Sigma) for one hour and washed to remove unbound IgG were added to BMDM at a 10∶1 ratio, centrifuged at 200× g at 4°C for 5 min to promote contact of the beads with the cells, and then incubated for varied lengths of time prior to fixation with 4% paraformaldehyde (PFA) and washing to remove unbound beads.

### Quantitation

Phagocytosis efficiency was analyzed using bright field microscopy at 40× magnification 40 min after the addition of opsonized beads to the BMDM, and the total number of cell-associated beads and the number of cells per field counted to determine beads/cell. Extracellular beads identified via fluorescent immunostaining with Cy-3 conjugated anti-human IgG (Sigma) prior to permeabilization of the cells were subtracted from total number of beads considered to be phagocytosed. A minimum of three fields at 63× magnification were counted for each experimental sample and then normalized to the wild-type (WT) control. Rac1 and DOCK2 localization were detected by immunofluorescence and visualized by confocal microscopy using a Leica SP5 instrument.

To assess distinct vs. indistinct cups, MATLAB was used to measure intensity, eccentricity, and completeness of the cups using output generated from the Leica software (script by James J. McCann, available on request). In brief, MATLAB generated ellipses based on mid-points of the minimal and maximal widths of the cup which were denoted by an investigator blinded to the experimental condition. Intensity was measured for each point on the ellipses line and summed. Eccentricity was calculated to determine how far the defined cup deviated from a perfect circle, and completeness was defined as the percent of points on the circle that were above background intensity. Distinct cups were defined as having an intensity of at least 12,000, an eccentricity of less than 0.3, and completeness of greater than 50%. Cups were quantitated from at least 3 fields at 63x magnification for a minimum of 3 independent BMDM isolations.

### Infection of BMDM with *Yersinia*

*Yersinia* strain IP2777c, a strain lacking the type III secretion system (T3SS) plasmid, was a kind gift from Dr. James Bliska and used as previously described [37]. Briefly, a multiplicity of infection (MOI) of 20 was used to infect mature BMDM. Following a 5 min centrifugation at 200× g at room temp to facilitate bacterial contact with the cells, samples were incubated at 37°C in 5% CO_2_ for 15 min. Bacterial uptake was measured by imaging at least 3 fields at 63× magnification using confocal microscopy.

### *In vitro* transwell migration assay

5×10^5^ blood neutrophils were placed in the top chamber of a 4 µm pore transwell apparatus (Corning) while the lower chamber was filled with media from either stimulated WT macrophages (LPS, 1 ug/mL for 2 hrs) or unstimulated WT macrophages. The apparatus was incubated for 30 min at 37°C in 5% CO_2_. Cells that migrated through the membrane were counted using a hemocytometer.

### Intraperitoneal (IP) white blood cell extraction

Mice were given IP injections of LPS (Sigma, 1 mg/kg) 4 hrs prior to sacrifice by cervical dislocation. Peritoneal white blood cells (WBC) were collected by PBS lavage of the peritoneal cavity followed by lysis of red blood cells. WBC were counted using a hemocytometer. To examine focal adhesions and F-actin defects, WBC were seeded on fibronectin-coated coverslips (BD transduction laboratories) and incubated at 37°C in 5% CO_2_ for 30 min and then stained for F-actin using rhodamine-phalloidin [38].

### Adhesion signaling

To measure podosome and focal adhesion signaling, 5×10^6^ bone marrow macrophages were serum starved at 37°C for 2 hrs and then seeded onto tissue culture 6-well plates and incubated for 30 min at 37°C in 5% CO_2_. The cells were collected in 2X Laemmli sample buffer, sonicated for 5 sec, and then boiled for 10 min (modified from [39]).

### PBD pull-down for activated Rac1

To measure activated Rac1, the p21 binding domain of PAK1 (PBD) fused with a GST tag was expressed in Bl21 cells and purified following methods previously described [8]. Cells were washed with phosphate-buffered saline (PBS) and lysed in ice-cold buffer (50 mM Tris, pH 7.6, 0.5 M NaCl, 0.1% SDS, 0.5% deoxycholate, 1% Triton X-100, 0.5 mM MgCl_2_, 0.2 mM sodium vanadate, 1 mM phenylmethylsulfonyl fluoride [PMSF], 1 μg/ml aprotinin, and 1 μg/ml leupeptin). Lysates were centrifuged at 14,000× *g* for 10 min at 4°C. Supernatants were incubated for 30 min at 4°C with 20 μg of glutathione-agarose beads coupled to glutathione transferase (GST)-PBD. Beads were sedimented and washed four times with 50 mM Tris-HCl, pH 7.6, containing 150 mM NaCl, 1% Triton X-100, and 0.5 mM MgCl_2_. Bound Rac proteins were detected by Western blotting, and whole cell lysates also were analyzed for Rac for normalization purposes.

### Macrophage and neutrophil recruitment to the pancreas during induction of acute pancreatitis

Acute pancreatitis was induced as previously described [40]. Briefly, 8-week old male mice were injected with 50 µg/kg caerulein (Sigma-Aldrich, St. Louis, MO) once per hour for seven hours. To assess recruitment, mice were sacrificed and the pancreata obtained one hour after the last injection. Pancreas sections were stained for neutrophils by performing IHC using Ly6B.2.

### Statistics

Numerical data are presented as mean ± SEM. Student's *t* test was used to compare the differences between two groups. Significance was judged when *P*<0.05.

## Results

### PLD1- and PLD2-deficient macrophages have distinct actin cytoskeleton defects

**Figure 1:**
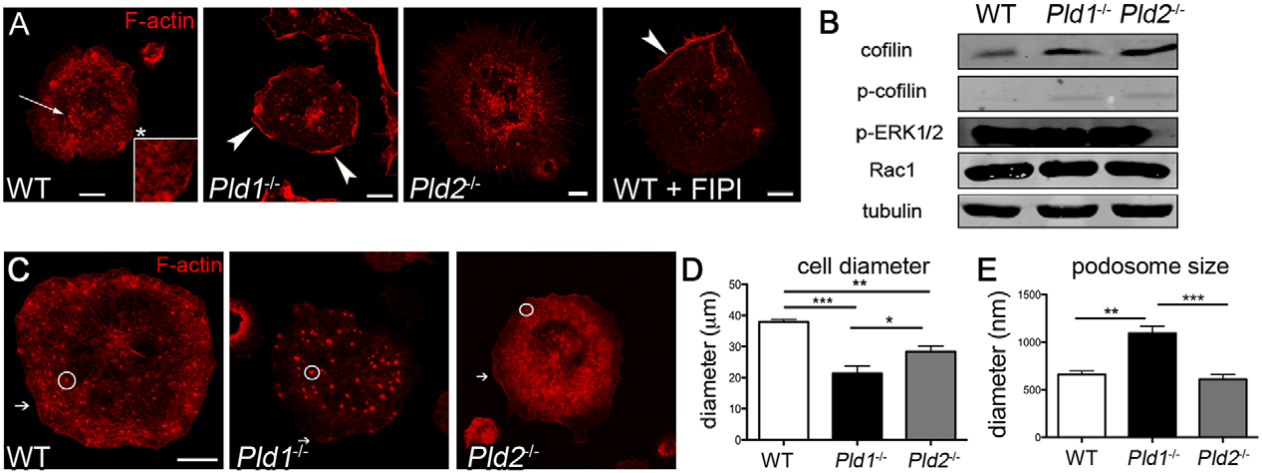
F-actin organization in resting and activated macrophages. A) Confocal images of BMDM transfected via nucleofection with the Spo20-GFP PA sensor (green), serum starved for 2 hrs, and then plated on fibronectin-coated coverslips for 30 min, fixed, and stained for F-actin using rhodamine phalloidin (red). Images are representative best of at least 3 separate experiments from which at least 3 macrophages were visualized. Arrow, podosome; arrowhead, cortical actin; *, region magnified in inset. B) Western blot analysis of BMDM macrophages treated as above. The experiment was repeated at least 3 times using BMDM prepared from different mice. C) Confocal images of intraperitoneal (IP) macrophages from WT, *Pld1* ^−/−^, and *Pld2* ^−/−^ mice serum-starved for 2 hrs and plated on fibronectin-coated coverslips. Macrophages were stained with rhodamine phalloidin to visualize F-actin. Podosomes are circled and arrows indicate cortical actin. Images are representatives of at least 3 experiments. D) Graph summarizing average cell diameter of IP macrophages on fibronectin. Data collected from at least 3 fields of vision from 3 independent mice. E) Graph representing the diameters of podosomes from IP macrophages plated on fibronectin. Data collected from at least 3 fields of vision from 3 separate mice. Bar, 7.5 µm. *  =  p<0.05, **  =  p<0.005, ***  =  p<0.0001.

Roles for PLD and PA in actin cytoskeleton reorganization have long been proposed [41] and the specific function of PLD in macrophages explored using macrophage-like cell lines [20], [42]. To examine F-actin organization in the absence of PLD isoforms or activity, we visualized the cytoskeleton of primary macrophages. Serum-starved, bone marrow-derived macrophages (BMDM) were generated and plated on fibronectin to enhance cell spreading (Fig. 1A). Wild-type (WT) BMDM exhibited an organized network of F-actin near the periphery (inset) and distinct adhesion structures known as podosomes (arrow), which play roles in directional movement [43]. In contrast, BMDM prepared from mice lacking PLD1 were more rounded and hence appeared smaller and more irregular in shape. F-actin was observed primarily at the cell cortex (arrowheads) in an exaggerated manner. Conversely, for BMDM prepared from mice lacking PLD2, F-actin manifested primarily in the perinuclear region, and virtually no cortical actin was observed. These findings raised a possibility that the isoforms exhibited partial redundancy, with each isoform partially compensating for the loss of the other. To test this possibility, we examined the cytoskeleton in WT BMDM pre-treated with 5-fluoro-2-indolyl des-chlorohalopemide (FIPI) [33]. FIPI directly inhibits PLD1 and PLD2 catalytic activity and has thus far not been reported to exhibit off-target effects [7], [30], [33], [44], [45], [46]. Macrophages were pre-treated with 750 nM FIPI, which results in total inhibition of PLD1 and PLD2 [33]. FIPI-treated BMDM appeared to have defects characteristic of both PLD1- and PLD2-deficient BMDM, wherein intense cortical actin was observed irregularly at the plasma membrane along with a loss of subcortical organized F-actin (Fig. 1A).

These differences lead us to test for differences in proteins involved in actin rearrangement. Western blot analysis indicated that *Pld1* ^−/−^ and *Pld2* ^−/−^ BMDM both have increases in cofilin, phospho (p)-cofilin (Fig. 1B), and paxillin (data not shown), which are actin turnover proteins involved in formation of focal adhesion sites such as podosomes [47]; there was no increase in Rac1, an F-actin organizing GTPase, or in p-ERK1/2, a cell growth/proliferation protein. Therefore, in addition to the localization defects observed in the BMDM membrane structures, PLD ablation appears to alter the expression of actin network proteins.

Actin structures and reorganization are important in cell motility. BMDM are not motile, and hence formation of podosomes, which are involved in macrophage movement [48], are infrequent in this setting. Having observed an increase in actin turnover proteins, we next wanted to assess the cytoskeleton in motile macrophages, for which we used activated macrophages collected from the peritoneum. Macrophages were recruited to the peritoneum by intraperitoneal (IP) injection of lipopolysaccharide (LPS), a potential activator of macrophages via Toll-like receptor stimulation, harvested by lavage, and plated on fibronectin. We found that for activated WT IP macrophages, F-actin formed a dispersed network of podosomes (Fig. 1C, circle indicates a representative podosome) and was localized at low but detectable levels at the cortex (arrow). The cells had an average diameter of 37.9+/−0.8 µm (Fig. 1D). The figures show representative images from at least three separate IP injections. At least 3 different cells from each mouse were visualized for quantitation. *Pld1* ^−/−^ macrophages also formed an organized network of podosomes, but the cells were 44% smaller than WT with an average diameter of 21.4+/−2.4 µm (p<0.0001). Most of the F-actin was found in the podosomes, and little to no cortical actin (arrow) was observed. *Pld2* ^−/−^ macrophages had visible cortical actin similar to WT cells (arrow), but spread only 28.4+/−1.8 µm and the podosomes were largely indistinguishable from the surrounding F-actin. *Pld1* ^−/−^ IP macrophage podosomes had diameters of 1093+/−75.3 nm, which was significantly larger than both WT (p<0.005) and *Pld2* ^−/−^ (p<0.0001) podosomes that had diameters of 658.8+/−37.6 nm and 609.9+/−49.7 nm, respectively (Fig. 1E).

The cell size differences, altered appearances of F-actin localization, and changes in podosome sizes in IP macrophages, in addition to the changes in expression level of actin-interacting proteins by Western blot analysis, indicate impairment in actin networking when either PLD isoform is deleted, although the underlying mechanisms are seemingly complex and vary from setting to setting. Taken together, our findings suggest that both PLD isoforms play a role in the maintenance of normal cytoskeletal appearance and PLD-generated PA production is important for regulating F-actin polymerization and cytoskeletal reorganization.

### Neutrophils lacking PLD isoforms exhibit migratory defects

**Figure 2:**
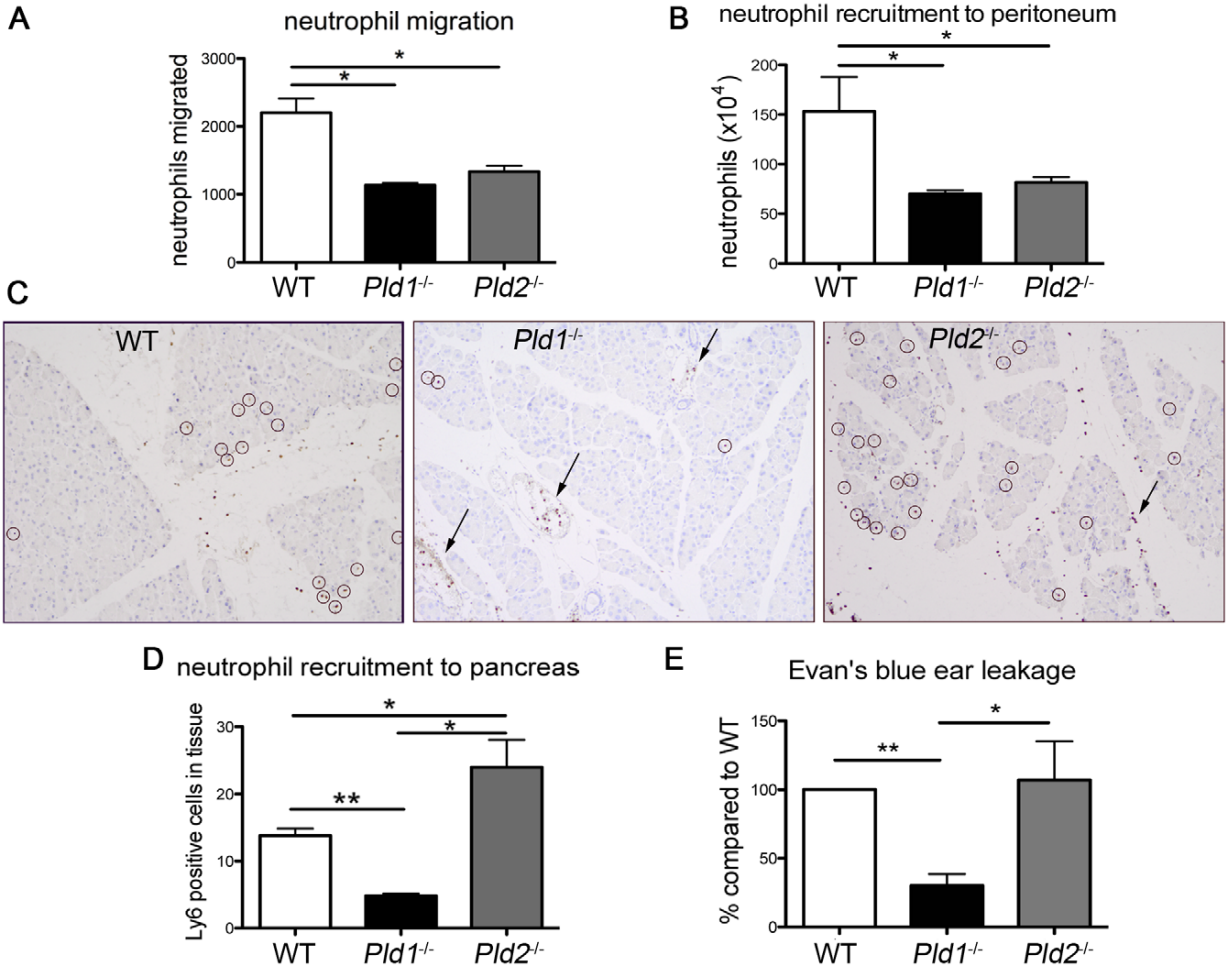
*Pld1* ^−/−^ and *Pld2* ^−/−^ neutrophils exhibit impaired migration but only *Pld1* ^−/−^ neutrophils have impaired tissue extravasation. A) Quantitation of chemoattractant-stimulated *in vitro* migration through 4 µm pore transwell filters of neutrophils isolated from the blood of WT, *Pld1* ^−/−^ and *Pld2* ^−/−^ mice. WT macrophages stimulated with LPS (1 µg/mL for 2 hrs) were used to generate conditioned media that was used as the chemoattractant. Blood neutrophils were allowed to migrate towards the conditioned media for 30 min. The experiment was performed at least 3 times using independently prepared BMDM. B) Quantitation of circulating neutrophil recruitment to the peritoneum. LPS (1 mg/kg) was injected into the peritoneum 4 hrs prior to sacrifice. The peritoneum was lavaged using HBSS to collect the recruited neutrophils; n = 3 for each type. C) IHC of pancreata stained with Ly6B.2 to detect neutrophils (circled) following induction of pancreatitis. Arrows indicate blood vessels and fibrotic border. D) Quantitation of neutrophil recruitment to the pancreas following acute pancreatitis. WT, n = 9; *Pld1* ^−/−^, n = 5; *Pld2* ^−/−^, n = 7. E) Quantitation of Evan's blue dye leakage into the ear following irritation with mineral oil. Results are the average of 3 independent experiments. *  =  p<0.05, **  =  p<0.005.

Dynamic rearrangement of actin is required for macrophages and neutrophils to migrate to sites of injury. As we observed defects in actin cytoskeletal structures including podosomes, which are necessary for motility, for both *Pld1* ^−/−^ and *Pld2* ^−/−^ macrophages, we next set out to examine if these defects affected cell migration. Neutrophil migration is more robust over short periods of time than macrophage migration and was thus chosen to explore potential migratory defects both *in vitro* and *in vivo*. The *in vitro* experiments employed conditioned media prepared from WT BMDM stimulated with LPS as a chemoattractant for blood neutrophils placed on a transwell filter. We observed that approximately half as many *Pld1* ^−/−^ (p<0.05) and *Pld2* ^−/−^ (p<0.05) neutrophils migrated towards the conditioned media as compared to WT neutrophils (Fig. 2A). For *in vivo* experimentation, we injected LPS intraperitoneally to induce neutrophil migration. As seen in our *in vitro* model, only half as many *Pld1* ^−/−^ (p<0.05) and *Pld*2^−/−^ (p<0.05) neutrophils migrated to the peritoneum (Fig. 2B).

We next tested the ability of *Pld1* ^−/−^ and *Pld2* ^−/−^ neutrophils to be recruited to sites of injury. Acute pancreatitis was induced through consecutive IP injections of caerulein, a cholecystokinin (CCK) analog that causes activation of digestive enzymes in the pancreatic epithelium resulting in inflammation, edema, and cell death. Mice were injected once/hour for 7 hours and allowed to recover for one or three hours, sacrificed, and their pancreata excised, fixed, and examined immunohistochemically for neutrophil migration. In WT mice, extravasation of neutrophils into the pancreatic parenchyma was readily apparent (Fig. 2C, D; circles indicate neutrophils). However, most of *Pld1* ^−/−^ neutrophils present did not appear to have entered the tissue, but rather were seen at areas of fibrosis or ductal borders (arrows), suggesting that *Pld1* ^−/−^ neutrophils have difficulties transversing into the interstitial tissue. Quantitatively, *Pld1* ^−/−^ pancreatic tissue had a significant reduction in neutrophil number within the tissue as compared to WT tissue (Fig. 2D, p<0.005). *Pld2* ^−/−^ mice, on the other hand, had a significant increase in the number of neutrophils recruited into the pancreas as compared to WT mice (Fig. 2D, p<0.05). *Pld2* ^−/−^ neutrophils were found throughout the tissue and blood vessels (Fig. 2C, right panel; arrow indicates blood vessel neutrophils). Since *Pld1* ^−/−^ and *Pld2* ^−/−^ mice exhibited such different responses *in vivo*, we then used an Evan's blue leakage assay to test vascular permeability during inflammation. We found that *Pld1* ^−/−^ mice had reduced vascular permeability (Fig. 2E), potentially explaining their confinement to non-interstitial tissues. Together, these data indicate that *Pld1* ^−/−^ and *Pld2* ^−/−^ neutrophils both have altered patterns of migration in response to chemokines in culture and/or to sites of injury *in vivo*, and that these defects, like the F-actin abnormalities, are isoform-specific and mechanistically complex.

### Macrophages lacking PLD1, PLD2, or all PLD activity have impaired phagocytosis

**Figure 3:**
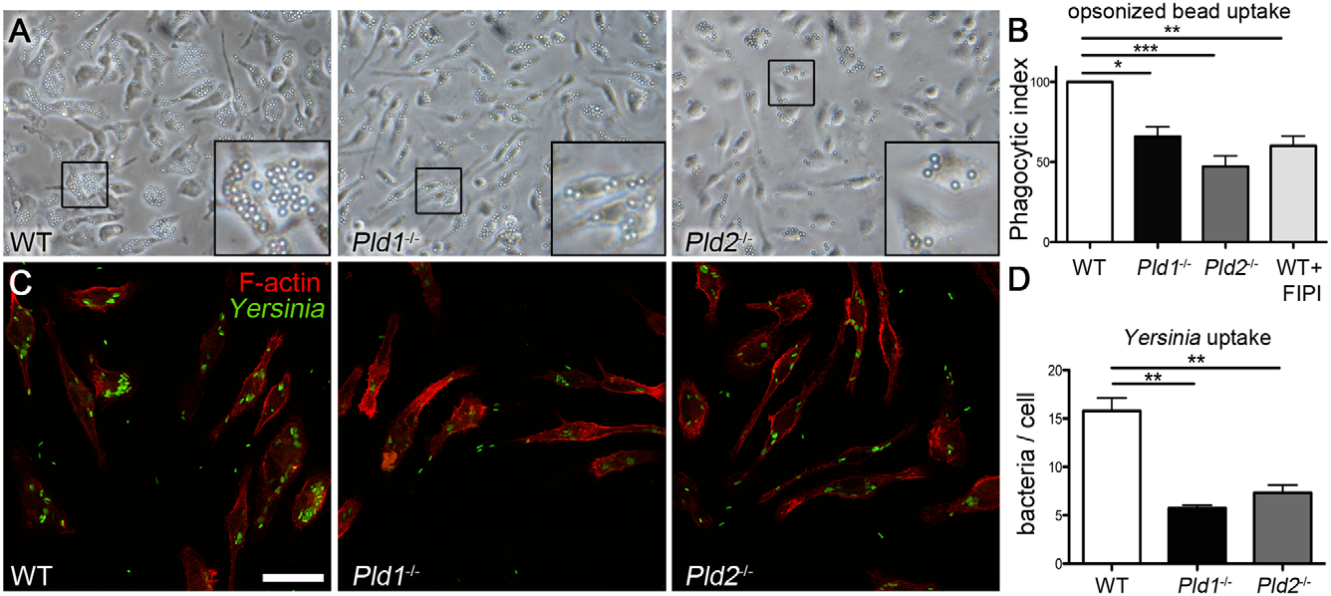
Loss of either isoform of PLD decreases phagocytosis. A) 40x brightfield images of BMDM cultured with human IgG-coated beads for 5 min and then fixed following washes to remove unbound beads. B) Graph of phagocytic index for WT, *Pld1* ^−/−^, and *Pld2* ^−/−^ BMDM as well as WT BMDM treated with FIPI (750 nM, pre-treated for 1 hr). Number of beads/cell was calculated as total number of beads/field divided by total number of cells/field. Index was defined as % of beads/cell phagocytosed by untreated WT BMDM. Extracellular (bound but not phagocytosed) beads were visualized by Cy5-anti human IgG immunostaining and excluded from the quantitation. Three fields were quantitated for each condition/day and the experiment was performed at least 5 times with BMDM prepared from different mice. C) Confocal images of WT, *Pld1* ^−/−^, and *Pld2* ^−/−^ BMDM that were cultured with GFP-expressing *Yersinia pseudotuberculosis* strain IP32777c at an MOI of 20 for 20 min, washed, fixed, permeabilized, and stained with rhodamine phalloidin (red). Images are ones typical of at least 3 experiments. Bar, 25 µm D) Graph of IP32777c uptake. Bacteria/cell was calculated as above for beads/cell. p-values: *  =  p<0.05, **  =  p<0.005, ***  =  p<0.0001.

Our results thus far suggested that the elimination of isoform specific PLD-generated PA results in abnormal cytoskeletal structures leading to impaired migration. Since phagocytosis is a temporally- and spatially-specific actin rearrangement process that shares many similarities with cell adhesion [25], we next examined if phagocytosis was affected. Phagocytosis by BMDM via the Fcγ receptor-mediated internalization pathway was assessed using IgG-coated beads. WT BMDM typically internalized 20–25 beads per cell (Fig. 3A). The precise average obtained in each experiment varied due to temperature, timing, and the state of the macrophages. To compare data generated from multiple experiments, the average number of beads phagocytosed by WT cells in an individual experiment was defined as 100% (Fig. 3B) and the averages of each other type of macrophage presented as a % of the WT uptake of beads. BMDM prepared from mice lacking PLD1 or PLD2 phagocytosed beads less efficiently (Fig. 3A, B), with a 34%±10% (p<0.05) decrease seen for *Pld1* ^−/−^ BMDM and a 57%±17% (p<0.0001) decrease for *Pld2* ^−/−^ BMDM. To test the possibility that the isoforms exhibited partial redundancy and each isoform partially compensating for the loss of the other, we assessed phagocytosis in WT BMDM pre-treated with 750 nM FIPI to block the activity of both PLD1 and PLD2. FIPI-inhibited macrophages internalized 40%±15% (p<0.005) fewer beads than untreated controls (Fig. 3B). These findings confirm and extend earlier reports that PLD activity is important in phagocytosis of IgG-opsonized targets, but also show that PLD1 and PLD2 undertake functions that are both non-redundant and non-additive, and that a basal level of phagocytosis persists even in the absence of all PLD activity. These results also implicate PLD activity, rather than PLD acting as a scaffold in the phagocytic process, since the PLD proteins were present but catalytically-inactive in the FIPI-treated cells.

Phagocytosis can also proceed via engagement of other types of cell-surface receptors that use and activate different signaling pathways and mechanisms [28]. For example, uptake of *Yersinia*, a gram-negative bacteria, occurs through binding of the bacteria to β1-integrin receptors, which triggers outside-in signaling. We thus infected WT, *Pld1* ^−/−^, and *Pld2* ^−/−^ BMDM with *Yersinia pseudotuberculosis* IP2777c, a strain easily phagocytosed by β1-integrin receptor uptake [49]. Similar to what was observed for IgG-coated beads, *Yersinia* phagocytosis was decreased 64% in the absence of PLD1 and 54% in the absence of PLD2 (Fig. 3C, D). Taken together, these findings suggest a general phagocytic defect in the absence of PLD1 and PLD2 that manifests downstream of receptor binding.

### Loss of PLD isoform activity results in dysmorphic phagocytic cups

**Figure 4:**
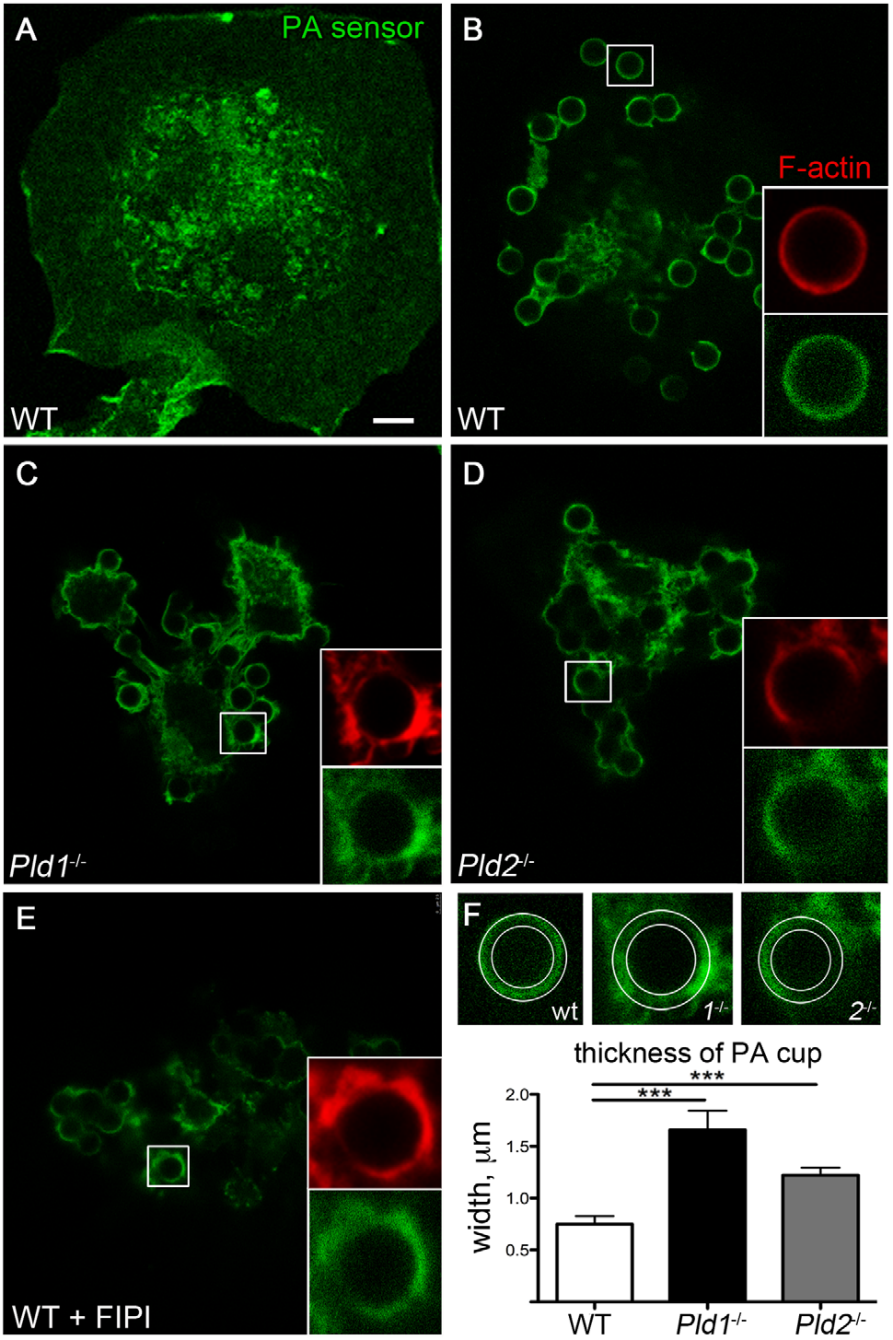
PLD deletion results in abnormal phagosomal cup formation. Confocal microscopy of primary BMDM transfected via nucleofection with an EGFP-fused Spo20-PA sensor to image PA (green) in unstimulated WT BMDM (A) or in WT (B), *Pld1* ^−/−^ (C), *Pld2* ^−/−^ (D), or FIPI-treated WT (E) BMDM that were cultured with opsonized beads for 5 min. F-actin was visualized using rhodamine phalloidin (red). Scale bar, 5 µm. Best representative images from at least 3 sets of independently-isolated BMDMs. F) Circles shown indicating thickness of cup in WT BMDM superimposed on *Pld1* ^−/−^ and *Pld2* ^−/−^ cups, with quantitation of the thickness of the PA-visualized cups. At least 10 cups were measured for each experimental setting, and the experiments were performed 3 or more times. ***  =  p<0.0001.

Taking advantage of the slower kinetics of IgG-coated bead phagocytosis, we next examined the phagocytic pathway in more detail. BMDM were transfected with the PA-binding domain of the yeast protein Spo20 fused to EGFP, which we have previously used as an *in vivo* sensor to visualize subcellular localization of PA [33]. Prior to being challenged with opsonized beads, the PA sensor localized in WT BMDM in a diffuse manner throughout the cell, with limited areas of concentration at the plasma membrane and the majority of the sensor in the nucleus (Fig. 4A). After addition of opsonized beads and progression of phagocytosis for 5 min, most of the PA sensor was observed to have translocated to the phagocytic cups (Fig. 4B). In WT BMDM, the phagocytic membranes, as imaged using the PA sensor, were zippered closely around the beads, forming a distinct cup of uniform intensity (Fig. 4B, inset) with a thickness of 0.75±0.2 µm (Fig. 4F). F-actin localization closely matched that of the PA-containing membranes, consistent with general models of phagocytosis in which the phagocytic process is driven by cortical F-actin assembly at the phagocytic membranes. In contrast, phagocytic cups in macrophages differentiated from *Pld1* ^−/−^ bone marrow were characterized by uneven localization of the PA sensor, resulting in irregular and broad cups (Fig. 4C) that were 1.66±0.5 µm in average width (p<0.001, Fig. 4F). F-actin distribution was similar (inset), suggesting that the PA sensor was localized to the surrounding phagocytic membranes. Phagocytic cups in macrophages differentiated from *Pld2* ^−/−^ bone marrow were also abnormal (Fig. 4D, F; 1.2±0.2 µm average width, p<0.001). These findings confirmed that both PLD1 and PLD2 participate in the phagocytic process and extends the findings by demonstrating that the phagosomes that do form in macrophages lacking either isoform are frequently abnormal. Since phagocytosis still occurred to some extent even when both isoforms of PLD were inhibited using FIPI (Fig. 3), we examined whether PA was generated by another source at the nascent phagosome, e.g. via a DAGK isoform [18], or whether phagocytosis could proceed, albeit with reduced efficiency, in the absence of PA generation. We thus pretreated WT BMDM with FIPI for one hour, allowed phagocytosis to proceed for 5 min, and examined PA localization. FIPI-treated WT BMDM imaged using the PA sensor exhibited broad, diffuse cups surrounding the phagocytosed beads (Fig. 4E) similar to those observed for the *Pld1* ^−/−^ and *Pld2* ^−/−^ BMDM. This result demonstrates that PA is generated at the phagocytic site in the absence of PLD1 and PLD2 by other enzymatic sources, but that in the absence of PLD1 and PLD2, the PA biogenesis occurs in an uncoordinated or altered manner that is disruptive to the normal phagocytic process.

### Altered localization of GTPases and GEFs that direct F-actin re-organization in macrophages lacking PLD activity

**Figure 5:**
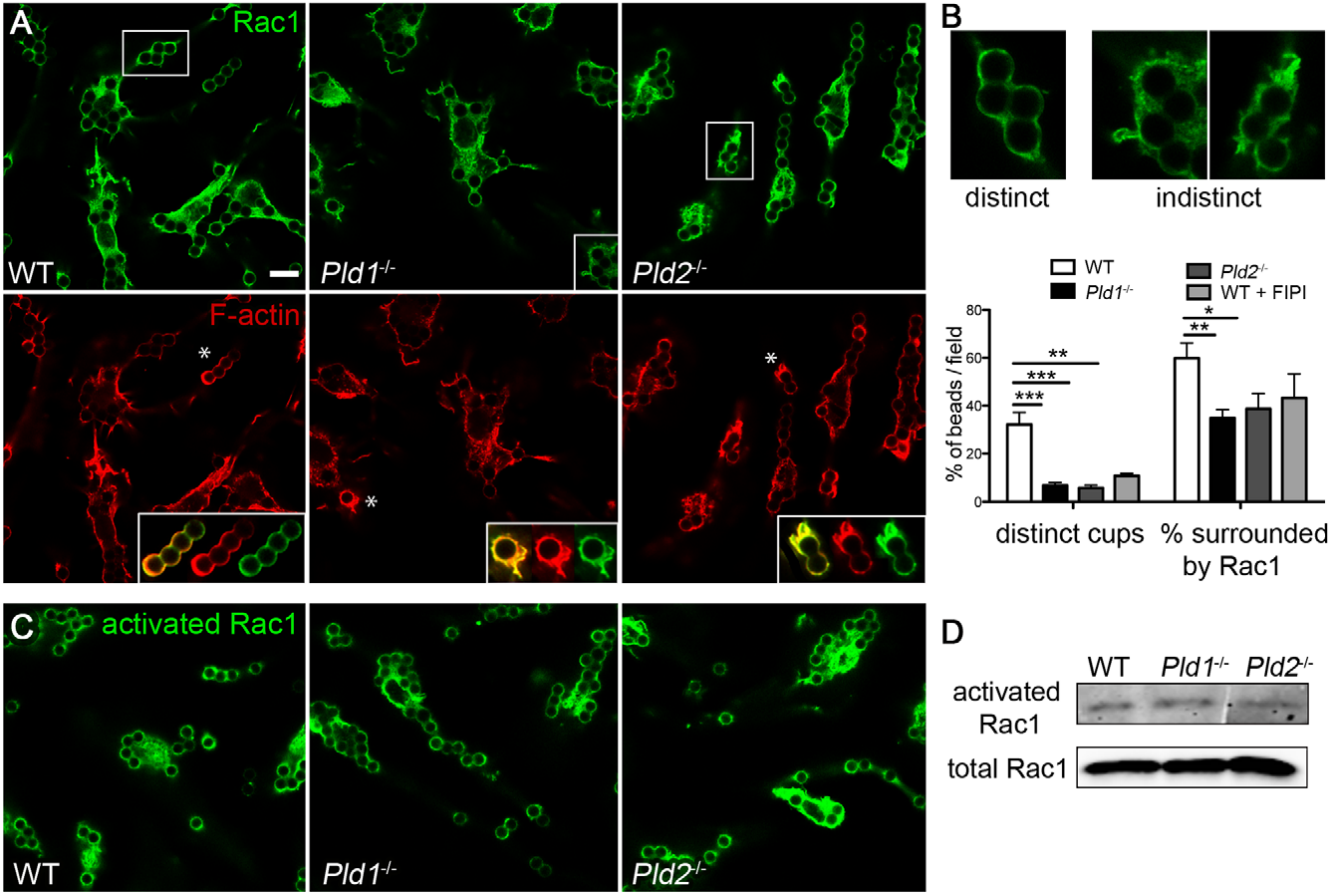
Rac1 recruitment and localization is abnormal in the absence of PLD1 or PLD2. A) Confocal microscopy of WT, *Pld1* ^−/−^, and *Pld2* ^−/−^ BMDM stimulated with human IgG-coated beads for 5 min followed by immunostaining for Rac1 (green) and staining for F-actin (rhodamine phalloidin, red). *, shown magnified in inset. B) Graph of distinct (example: top panel, left) and indistinct (examples: top panel, right) Rac1 cups. Type of cup (distinct versus indistinct) was normalized to the number of beads per field. Quantitation was performed using Zeiss intensity maps as well as MATLAB parameters as described in Materials and Methods. At least 3 fields and 20 cups were scored for each condition from 5 independent experiments. C) Confocal microscopy using an activation state-sensitive anti-Rac1 monoclonal antibody to visualize activated Rac1 in WT, *Pld1* ^−/−^, and *Pld2* ^−/−^ BMDM stimulated with human IgG-coated beads for 5 min. D) Western blot of PBD-pull down assay to assess GTP-bound Rac1 in WT, *Pld1* ^−/−^, and *Pld2* ^−/−^ BMDM stimulated with human IgG-coated beads for 5 min; total Rac1 in the lysates is shown as a loading control. The western was repeated at least 3 times with similar results. Bar, 7.5 µm. *  =  p<0.05, **  =  p<0.005, ***  =  p<0.0001.

Small GTPases such as Cdc42 and Rac1 are recruited during phagocytosis to participate in actin rearrangements [50]. Cdc42 activation has been observed early on in pseudopodia extension while Rac1 and Rac2 activation were observed in phagocytic cups and during phagosome closure [51]. Since some GTPases are known to posses PA binding sites and to be recruited by PA [5], [8], we next hypothesized that either Cdc42 or Rac1 might be mislocalized if the PA production at the phagosome was altered. To test this, macrophages were stimulated with opsonized beads for 5 min and then immunostained for Cdc42 and Rac1. Only low levels of Cdc42 were detected at the WT phagosome (data not shown). Rac1, on the other hand, formed clearly visible and well-formed phagocytic cups around the newly ingested beads in WT BMDM (Fig. 5A). However, the quantity and quality of the cups were reduced in *Pld1* ^−/−^ and *Pld2* ^−/−^ BMDM as well as in WT BMDM pre-treated with FIPI to inhibit both isoforms.

To quantitate localization of Rac1 to the phagosome, we classified the Rac1 cups surrounding the beads as either distinct (Fig. 5B, left panel) or indistinct (Fig. 5B, right panel), using the MATLAB parameters described in Materials and Methods. *Pld1* ^−/−^ and *Pld2* ^−/−^ BMDM had a greatly reduced number of distinct cups, 19% and 16%, respectively, of that observed in WT BMDM (Fig. 5B, left graph). Additionally, 60% of the beads in the WT BMDM were surrounded by Rac1 to at least some extent, whereas that was the case for only 34% of the beads in *Pld1* ^−/−^ BMDM and 40% of beads in *Pld2* ^−/−^ BMDM, indicating not only a reduction in Rac1 recruitment (Fig. 5B, right graph), but also an imprecision in localization once at the site. Pharmacological blockade of both PLD isoforms by FIPI resulted in similar decreases in formation of distinct cups and Rac1 recruitment.

Only GTP-bound Rac1 can interact with the effector proteins WAVE, WASP, or Arp2/3 to stimulate actin polymerization. If PLD activity exerted a substantial effect on the GTP vs GDP bound state of Rac1, there could be less activated Rac1 in the PLD-deficient macrophages, leading to less actin polymerization and phagocytosis. To test this possibility, we used an activation-specific anti-Rac1 monoclonal antibody to examine the localization of GTP-Rac1 as well as a PBD (the GTP-Rac1-binding domain of PAK1) pull-down assay to assess the amount of GTP-bound Rac1. Activated Rac1 was observed to surround the beads in macrophages prepared from all three strains of mice (Fig. 5C), although both *Pld1* ^−/−^ and *Pld2* ^−/−^ BMDM exhibited a higher frequency of cups with uneven Rac1 localization. PBD pull-down demonstrated no difference in the amount of activated Rac1 in the PLD-deficient BMDMs (Fig. 5D).

**Figure 6:**
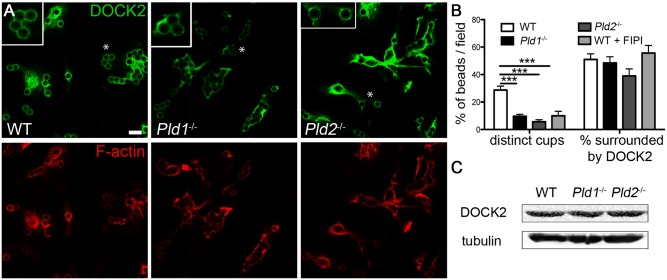
DOCK2 mislocalizes in the absence of PLD1 or PLD2. A) Confocal microscopy of WT, *Pld1* ^−/−^, and *Pld2* ^−/−^ BMDM stimulated with human IgG-coated beads for 5 min followed by immunostaining for DOCK2 (green) and rhodamine phalloidin staining of F-actin (red); *, region magnified in inset. B) Graph of distinct and indistinct DOCK2 cups as well as actin cups, quantitated as in Fig. 2. C) Western blot of DOCK2 in WT, *Pld1* ^−/−^, and *Pld2* ^−/−^ BMDM, best representative images from at least 3 blots. Bar, 7.5 µm. ***  =  p<0.0001.

The DOCK2 GEF exchanges GDP for GTP on Rac1 and also possess a binding site for PA that facilitates its recruitment to the plasma membrane at sites of signaling during chemotaxis [7], [52]. To examine potential DOCK2 recruitment to the phagosome, localization studies were performed as for Rac1 above. Similar as for Rac1, a decrease in the relative enrichment of DOCK2 surrounding the beads and a patchy distribution of DOCK2 at the bead surface was observed in the absence of PLD isoforms or activity (Fig. 6A). 29% of the phagocytic cups in WT BMDM were distinct cups, whereas only 10% (p<0.005) of the *Pld1* ^−/−^ BMDM cups were distinct, and 6% (p<0.005) were distinct in the *Pld2* ^−/−^ BMDM (Fig. 6A, B). Although *Pld2^−/−^* BMDMs appear to express higher levels of DOCK2 in some cells in the field shown in Fig. 6A, Western blot analysis revealed no difference in the amount of DOCK2 protein in the BMDM from the different strains (Fig. 6C). These results indicate that DOCK2 is recruited to the phagosome during IgG-receptor internalization events but its localization is abnormal in the absence of PLD isoforms or activity, leading to altered Rac1 recruitment and its mislocalization.

Taken together, our findings show that loss of PLD results in dysregulation of actin reorganization in an isoform-specific manner and that these changes have effects on immune cell migration and phagocytosis in culture and *in vivo*.

## Discussion

Roles for Phospholipase D in phagocytosis and neutrophil migration have been proposed based on intriguing findings made using imperfect tools such as 1-butanol, a crude PLD inhibitor with off-target effects; RNAi, which does not completely eliminate PLD expression; and macrophage-like transformed cell lines, which are excellent models but not completely identical to primary cells in their behaviors [20], [21]. Here, we describe use of primary cells from mice lacking PLD1 or PLD2 activity, the small molecule PLD inhibitor FIPI, and *in vivo* model systems to examine actin reorganization and immune cell function in the absence of PLD1 or PLD2 or pharmacological inhibition of both isoforms. Our findings both support the prior studies and reveal a number of new findings.

Our first observation was that the actin structures found in *Pld1^−/−^* and *Pld2^−/−^* macrophages (and neutrophils, not shown) are notably different. Although actin dynamics are complex and the phenotypes seen varied according to the setting, a general finding was that in the absence of PLD1, we saw exaggerated F-actin polymerization at the cell cortex and at sites of attachment to extracellular substrates, whereas in the absence of PLD2, such peripheral F-actin was diminished and central F-actin organization predominated. The exaggerated F-actin polymerization seen in the cellular periphery in the absence of PLD1 suggests that PLD1 plays a role, at least in part, to inhibit PLD2 or another source of PA at the plasma membrane. This idea is supported by the diminishment of cortical actin in the absence of PLD2. It might seem contradictory at first that the two isoforms display seemingly opposite phenotypes, but PLD2 has been observed to localize to the plasma membrane and to have higher basal activity than PLD1 [53], [54], and there is precedent for these differences. Our findings echo observations made during the initial cloning of the PLD genes and microinjection of PLD cDNAs into cells to examine the transient effects on the cytoskeleton [53] that have been confirmed and extended subsequently [16], [54], [55], although not without findings that suggested converse roles for the isoforms as well [55], [56].

Several mechanisms might underlie these findings. First, in both knock-out genotypes, we observed an increase in both cofilin and phosphorylated cofilin, which might prolong or exaggerate other actin defects. Cofilin interacts with both G and F actin and increases the number of barbed ends of growing filaments, resulting in actin severing and turnover. However, the net result on the cell depends on the concentration of these populations. Moreover, phosphorylation of cofilin leads to its inactivation [57] which presumably stabilizes actin structures. Differences in the subcellular localization of the increased cofilin and phospho-cofilin might causes the actin structures to turn over more quickly or slowly. Studying podosome turnover time may provide further insights.

Alternately, podosomes share many structural and regulatory elements with focal adhesions [58], which undergo assembly and disassembly via endocytosis and exocytosis mechanisms [59] that we have previously shown to be facilitated by PLD1 [60], [61] and PLD2 [54]. Finally, podosome assembly and disassembly has also been reported to be regulated by integrin α_v_β_3_ signaling, another pathway that we have identified roles for PLD1 in through the study of PLD1-deficient mice and cells [30], [34].

Although the specific details of the actin defects were different between *Pld1^−/−^* and *Pld2^−/−^* macrophages, they were both abnormal. It is therefore unsurprising that this lack of precisely controlled actin regulation led to decreased cell motility *in vitro* as we observed for deficiency of each isoform. However, how these findings play out *in vivo* is more complicated. While neutrophils from both knockout genotypes were less motile in vivo in the context of recruitment to the peritoneum, a specific inability of *Pld1* ^−/−^ neutrophils to migrate into injured pancreatic tissue was observed and may be more attributable to the restricted vascular permeability we also found for the mice.

Adhesion hubs and phagocytosis share many similar components [25]. Defects in podosomes and motility were therefore a good indication that phagocytosis would also be impaired in the absence of PLD1 and PLD2 as has been previously reported [20], [21], [42]. In line with these reports, we did find that both PLD1 and PLD2 are required for the normal progression of phagocytosis. Most significantly, what we found is that in the absence of either isoform, phagocytosis is blunted, but does not completely cease and that PA is still found on the phagosome. This might in theory signify partial redundancy in function for the isoforms. However, no further decrease in phagocytosis was seen when both isoforms were pharmacologically silenced, suggesting instead that PLD1 and PLD2 act at distinct steps of a phagocytic pathway that can still function, albeit inefficiently, in their absence. The major effects of loss of PLD gene function or activity appear to be on the relationship of the zippering membranes to the phagocytosed object. Regardless of whether we imaged a signaling or organizing protein (e.g. Rac1, Dock2, or F-actin) or a signaling lipid (PA), we saw “thickened” and irregular phagocytic cups. This appearance suggests a ruffling phenomenon wherein the membranes are poorly zippered in apposition to the object being phagocytosed, leading to membrane undulations that give the impression of thickened membrane structures within the resolving power of the confocal microscope. Thus, although other sources of PA suffice to form an F-actin structure that creates a phagocytic cup, its integrity and the final, refined structure depends on PLD-generated PA.

Surprisingly, we found that PA is still generated at the phagocytic cup when both isoforms are inhibited. There are two other pathways through which PA can be generated – lysophosphatidic acetyltransferase (LPAAT) and diacylglycerol kinase (DAGK). There are many LPAAT isoforms, some of which have been associated with membrane vesicle trafficking processes including endocytosis [56], [62]; however, none have been studied in the context of phagocytosis. In contrast, DAG is well known to play important roles in phagocytosis and downstream effector pathways [63], and DAGK has been found at the phagocytic cup although it is not known whether its function there is enzymatic or as a scaffold [18]. Our findings suggest that DAGK isoforms, and possibly LPAATs, may produce PA during phagocytosis, although seemingly not with sufficient accuracy in timing, location, and/or amounts to fully compensate for the loss of either PLD isoform.

Ultimately these studies highlight the need for continued effort to gain mechanistic insights into the roles that PLD isoforms and PA play in immune cell function.
